# Characteristics of horse riding-related falls in patients presenting to emergency departments in manche department, france: a multicenter retrospective analysis

**DOI:** 10.1186/s13049-026-01593-6

**Published:** 2026-03-10

**Authors:** Juliette Lemercier, Jeremy Pasco, Grégoire Olivier, Félix Amiot

**Affiliations:** 1 Emergency Department-SAMU50, Centre Hospitalier Mémorial Saint-Lô, Saint-Lô, France; 2 Clinical Research and Innovation Unit, Centre Hospitalier Public du Cotentin, Cherbourg-en-Cotentin, France

**Keywords:** Horse riding-related injuries, Incidence, Traumatic brain injury, Spinal injury, Prevention

## Abstract

**Background:**

Horse riding-related falls are a frequent cause of emergency department (ED) presentations; however, their regional epidemiology in France is poorly described. We aimed to quantify the incidence of ED visits after falling from a horse in the Manche Department and characterize the injury patterns, resource use, and short-term outcomes.

**Methods:**

We conducted a multicenter retrospective study across six hospitals in Manche (France), including all patients presenting to the ED after a fall from a horse between May 1, 2021, and October 31, 2024. Demographics, initial clinical status, imaging findings, anatomical injuries (Abbreviated Injury Scale [AIS]), management, and use of protective equipment were extracted from the electronic records. The incidence was standardized according to the catchment population of each center. Reporting was performed in accordance with the Strengthening the Reporting of Observational Studies in Epidemiology guidelines.

**Results:**

This study included 669 ED visits after falling from horses. The average annual incidence was 93.6 per 100,000 inhabitants, with substantial geographical variation. Most patients were women (87%), with a median age of 18 years. Spinal (43%), head (39%), and upper limb (33%) injuries were most common. Helmet use was high (97%) and likely contributed to the low rate of severe intracranial injuries. Imaging was performed in 93% of the patients, with 42% showing abnormalities. Most patients (80%) were discharged home, whereas 18% required hospitalization (median stay, 2 days), and 8.5% required surgery, mainly for fractures.

**Conclusions:**

Horse riding-related falls are a prevalent cause of ED visits in the Manche region, with substantial geographic variation in incidence. Although most injuries are minor, the high frequency of head and spine involvement underscores the importance of protective equipment and targeted prevention strategies. Standardized imaging and discharge pathways can streamline resource utilization while ensuring the detection of clinically significant injuries.

**Trial registration:**

Not applicable (retrospective observational study).

**Supplementary Information:**

The online version contains supplementary material available at 10.1186/s13049-026-01593-6.

## Background

Equestrian activities, encompassing professional, recreational, and therapeutic practices (e.g., horse-assisted therapy), rank among those with the highest accident rates despite growing global popularity [1]. According to the International Equestrian Federation, the number of riders is increasing significantly and consistently each year [1]. The kinetic energy generated by the horse’s mass and speed makes falls inherently high-energy events, which substantially increases injury severity [2]. Head injuries, spinal fractures, and limb fractures are the most common consequences of equestrian falls [3]. Equestrian activities represent the leading cause of sports-related spinal and chest injuries, surpassing snowboarding, skiing, soccer, and weightlifting [4]. Most fatalities result from severe head trauma [5]. The literature indicates particularly high injury frequencies among children, young adults, women, and amateur riders, compared with professionals [6, 7]. In pediatric populations, horse riding-related injuries constitute a significant proportion of sports injuries overall [8]. Protective equipment, particularly helmets, reduces head injury severity, as supported by matched-pair analyses [9]. The role of other protective devices, such as air jackets, remains debated, with some evidence suggesting they may not reduce—and could potentially increase—the risk of severe injury in certain equestrian disciplines [10].

In France, horse riding is the third most popular sport after soccer and tennis, with approximately 670,000 licensed riders and nearly three million regular or occasional participants [11]. The Weekly Epidemiological Bulletin reports approximately 6,000 horse riding-related injuries in France annually, with seven fatalities [12]. French studies, such as Boiron et al. [13] on craniofacial trauma, have highlighted specific injury patterns, but regional epidemiologic data remain sparse [13]. These data underscore the public health challenges posed by equestrian injuries, particularly in regions with high levels of equestrian activity, such as Manche in Normandy [14].

Despite the well-established benefits of protective equipment demonstrated in international studies, few epidemiological studies have been conducted in France. In regions where horse riding is integral to culture and leisure, a lack of regional epidemiologic data impedes the development of evidence-based, geographically tailored preventive measures. Furthermore, many epidemiological studies have not differentiated between falls from horses and accidents occurring when the rider is on the ground (such as bites and kicks), despite fundamental differences in their mechanisms, severity, and consequences [15–17]. Falls from horses represent distinct high-energy mechanisms requiring separate clinical and epidemiologic analysis.

Therefore, we conducted a multicenter retrospective study of injuries resulting from horse falls presenting to emergency departments (EDs) in the Manche Department, France. Our specific objectives were: (a) to quantify the number of ED visits due to horse riding falls and estimate the incidence according to each participating center and its surrounding population; (b) to describe the demographic characteristics of the patients sustaining these injuries; (c) to describe the use and type of protective equipment at the time of injury; (d) to characterize the types and severity of injuries sustained; and (e) to describe hospital resource utilization and immediate clinical outcomes in affected patients.

## Methods

### Ethics approval

The study protocol was approved by the Local Ethics and Research Committee of the Caen University Hospital. Information and non-opposition notes were sent to the patients or the parents/legal guardians of minor patients; they had 30 days to express their refusal. In accordance with French law, no response was considered non-opposition. All data were anonymized in accordance with the Reference Methodology 004 (MR-004) of the Commission Nationale de l’Informatique et des Libertés.

### Study design

We conducted a multicenter retrospective study. Patients were selected at six community hospital (Cherbourg, Saint-Lô, Coutances, Granville, Avranches, Saint-Hilaire) in Manche, France. This area was selected because of its high density of horse riders. All six EDs in Manche were included, covering the entire department; no other EDs exist in the region.

### Settings

The Manche Department, located in Normandy, France, spans 5,938 km^2^ with a population of approximately 500,000 inhabitants, predominantly living in rural areas (70%) [18]. The region has a temperate oceanic climate and a high level of equestrian activity [19]. The French emergency system includes the Emergency Medical Assistance Service** (**SAMU) and the Mobile Emergency and Resuscitation Service (SMUR) services for prehospital care. Manche features six EDs with varying trauma capabilities, and no level 1 trauma center exists in this department.

### Reporting guideline adherence

This study was designed and reported in accordance with the Strengthening the Reporting of Observational Studies in Epidemiology statement.

### Inclusion period

Patients were included between May 1, 2021, and October 31, 2024. The start date corresponds to the end of the third coronavirus disease 2019 (COVID-19) lockdown in France (May 2021), when leisure activities, including horse riding, resumed. Analysis of complete calendar years was not possible due to multiple pandemic-related lockdowns that restricted access to equestrian facilities during 2020 and early 2021. The end date for data collection (October 31, 2024) corresponds to the study initiation date; this date was chosen to maximize the number of included subjects while maintaining the retrospective design. This period corresponds to the marked resumption of equestrian activities after the COVID-19 pandemic.

### Participants

#### Case identification and data collection from patient records

To identify patients meeting the inclusion criteria, a structured query was performed across all medical and paramedical ED records during the study period. The query searched for predefined keywords related to the exposure of interest, including fall, horse, horse riding, equestrian, rider, and their lexical variants. Subsequently, a manual review of the complete medical record was conducted for each screened patient to confirm eligibility and refine the final study population.

#### Inclusion criteria

Patients were eligible for selection, regardless of age, if they were treated in the ED for trauma directly related to a fall from a horse. A “horseback riding fall” was defined as any unintentional dismount from a horse or pony during mounted activity. Only falls were included and other equids (donkeys, mules) were not included.

#### Non-inclusion criteria

Patients were excluded if they (1) were pregnant, (2) experienced equestrian trauma without falling (e.g., horse-kick, bite), or (3) had records with missing data for essential variables (patients who left the ED without waiting for the results of additional tests, patients who were lost to follow-up, absence of initial clinical examination noted). Pregnant women were excluded because of the risks associated with radiation exposure from imaging and potential confounding due to pregnancy-related physiological changes. Non-fall mechanisms (e.g., kicks, bites) were excluded to focus on high-energy injuries sustained while mounted.

To ensure a comprehensive capture of horse riding-related falls in the Manche Department, we additionally reviewed all SMUR interventions during the inclusion period. This was performed to identify patients who were directly transported from the prehospital setting to an external trauma center (e.g., Caen University Hospital), bypassing the six participating community hospitals. The SMUR records were obtained from the Departmental Emergency Medical Services database and cross-referenced according to the study’s inclusion criteria. Patients who met the criteria but were transferred to external facilities were counted for reporting purposes but not included in the primary cohort, as their complete electronic medical records were unavailable at the study sites.

### Data collection

Information was extracted from the electronic medical records of the participating centers. After pseudoanonymization, the following data were collected: (a) sociodemographic data, including age, sex, height, and weight (body mass index calculation); (b) initial clinical status, including heart rate, blood pressure, oxygen saturation, Shock Index, and Glasgow Coma Scale (GCS) score; (c) mode of access to the ED, categorized as independent, ambulance, mobile emergency, and resuscitation service (SMUR) or referred by a general practitioner; (d) assessment of severity, including Injury Severity Score (ISS), Severe Trauma Network Classification (grades A–C), and AIS score; (e) type of injuries, including head trauma, fractures, dislocations, sprains, wounds, contusions, and internal injuries, and location, including head, face, spine, chest, abdomen, pelvis, limbs, and skin; (f) imaging findings and results, including radiography, computed tomography (CT), magnetic resonance imaging, and ultrasonography; (g) management-related data, including referral (home, hospitalization, rehabilitation, intensive care, or death), indication and nature of surgery, and length of hospital stay; and (h) protective factors such as wearing a helmet and other safety equipment.

The AIS and ISS score were determined sequentially: the AIS was calculated first based on the Traumabase group methodology [20]. The ISS score was then determined using the Medicalcul calculator available online (http://medicalcul.free.fr/issrtstriss.html).

### Statistical methods

Qualitative variables were described as numbers and proportions. Quantitative variables were described as medians and interquartile ranges (IQRs). Continuous variables are reported as median and IQR, and categorical variables as counts and percentages. Comparisons according to sex (two groups) were performed using the Pearson’s chi-squared test for categorical variables and the Wilcoxon rank-sum test for continuous variables. Comparisons across age groups (four categories) were conducted using Fisher’s exact test for categorical variables and the Kruskal–Wallis rank-sum test for continuous variables. All tests were two-sided, and a p-value < 0.05 was considered statistically significant. All analyses were performed using R software version 4.5.1 (R Foundation for Statistical Computing, Vienna, Austria).

## Results

### Study population selection (Fig. 1)

**Fig. 1:**
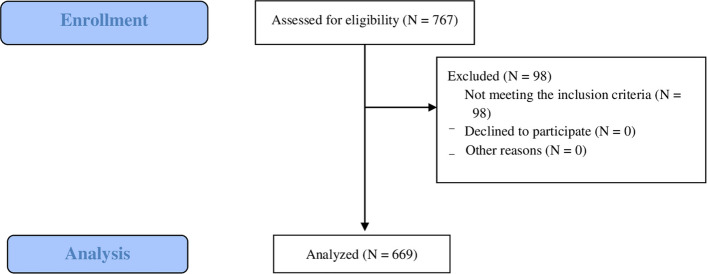
Flowchart

#### Population characteristics

We included 669 ED visits after a fall from a horse (May 2021–October 2024); 580 (87%) were women, and the median age was 18 years (IQR, 13–29). Most patients arrived independently (*N* = 442, 66%), 202 (30%) with ambulance, 19 (2.8%) with the SMUR, and six (0.90%) with general-practitioner referrals. Initial vitals were frequently normal; the GCS score was 15 in 99% of the visits. An additional seven patients were identified from SMUR records who were directly transported to an external trauma center (Caen University Hospital) without presentation to a study site ED. These patients were excluded from the primary analysis owing to the lack of accessible records but were noted to provide context for potentially missed severe cases.

**Fig. 2:**
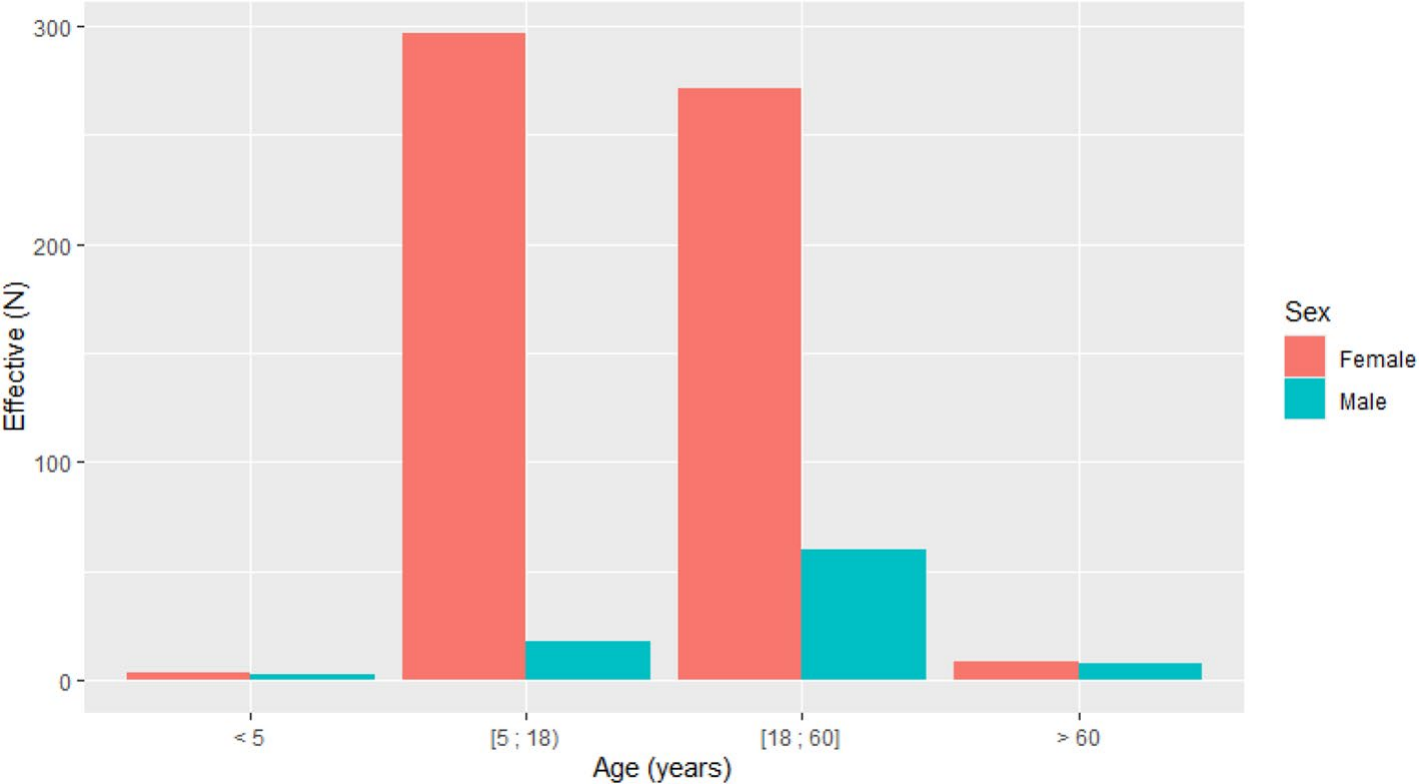
Distribution of patients by age group and sex (*N* = 669)

The median Shock Index was 0.71 (IQR, 0.61–0.81). The trauma network grade C was recorded for 55 patients among available data (*N* = 75), and no cases of grade A or B were identified. The age distribution comprised four groups: < 5 years (*N* = 7, 1.0%), 5–18 years (*N* = 314, 47%), 19–60 years (*N* = 331, 49%), and > 60 years (*N* = 17, 2.5%) (Fig. 2). Admission rates differed significantly across age groups: 65% of patients > 60 years required hospitalization, compared to 20% in the 5–18 and 19% in the 19–60 year groups (*p* < 0.001, Fisher’s exact test). Imaging utilization did not differ significantly across age groups (p = 0.066). Male riders (*N* = 89, 13%) tended toward higher admission rates (27% vs. 19%; *p* = 0.095, Pearson’s chi-squared test) and more frequent abnormal imaging findings (45% vs. 36%; *p* = 0.12). Overall demographic data, vital parameters, clinical examination findings, and injury details stratified by age group and sex are presented in Tables 1, 2, 3 and 4 and Supplementary Tables S1–S3.

**Table 1:** Demographic and clinical characteristics by sex and age group

Sex	Female				Male			
Age (years)	< 5 *N* = 4^1^	[5; 18) *N* = 296^1^	[18; 60] *N* = 271^1^	> 60 *N* = 9^1^	< 5 *N* = 3^1^	[5; 18) *N* = 18^1^	[18; 60] *N* = 60^1^	> 60 *N* = 8^1^
Mode of ED arrival								
Self-presented	4 (100%)	208 (70%)	166 (61%)	6 (67%)	3 (100%)	10 (56%)	41 (68%)	4 (50%)
MT General practitioner referral	0 (0%)	1 (0.3%)	5 (1.8%)	0 (0%)	0 (0%)	0 (0%)	0 (0%)	0 (0%)
Mobile Emergency & Resuscitation Service (SMUR)	0 (0%)	7 (2.4%)	8 (3.0%)	0 (0%)	0 (0%)	0 (0%)	3 (5.0%)	1 (13%)
Ambulance (fire/EMS, “SAV”)	0 (0%)	80 (27%)	92 (34%)	3 (33%)	0 (0%)	8 (44%)	16 (27%)	3 (38%)
Height (cm)	–	158 (148, 164)	165 (161, 169)	158 (158, 158)	–	145 (118, 165)	177 (174, 181)	182 (172, 191)
Missing	4	252	248	8	3	15	48	6
Weight (kg)	18 (15, 20)	45 (32, 55)	60 (55, 70)	64 (60, 65)	20 (20, 20)	31 (31, 33)	78 (64, 87)	73 (73, 105)
Missing	0	209	245	6	2	13	46	5
Body mass index (kg/m^2^)	–	19.0 (16.8, 20.9)	21.9 (20.6, 24.8)	25.6 (25.6, 25.6)	–	14.7 (13.6, 21.3)	24.3 (20.8, 27.2)	26.7 (24.7, 28.8)
Missing	4	252	249	8	3	15	48	6
Heart rate (bpm)	85 (85, 85)	89 (81, 101)	84 (73, 92)	88 (72, 97)	–	86 (80, 101)	73 (64, 82)	71 (60, 87)
Missing	3	124	71	3	3	6	17	1
Systolic blood pressure (mmHg)	112 (112, 112)	116 (107, 126)	121 (112, 130)	158 (135, 174)	–	114 (106, 123)	129 (118, 138)	147 (129, 153)
Missing	3	126	71	3	3	6	17	1
Diastolic blood pressure (mmHg)	61 (61, 61)	67 (61, 77)	73 (66, 81)	69 (56, 97)	–	61 (59, 66)	76 (67, 86)	81 (74, 101)
Missing	3	124	72	3	3	6	17	1
Oxygen saturation (%)	–	99 (99, 100)	99 (98, 100)	98 (98, 100)	–	99 (98, 100)	99 (97, 100)	97 (94, 99)
Missing	4	125	73	3	3	6	16	1
Shock index	0.76 (0.76, 0.76)	0.79 (0.70, 0.87)	0.69 (0.60, 0.77)	0.55 (0.46, 0.66)	–	0.77 (0.67, 0.96)	0.57 (0.51, 0.65)	0.55 (0.37, 0.62)
Missing	3	127	71	3	3	6	17	1
Glasgow Coma Scale								
13	0 (0%)	0 (0%)	1 (0.4%)	0 (0%)	0 (0%)	0 (0%)	0 (0%)	0 (0%)
14	0 (0%)	2 (0.7%)	0 (0%)	0 (0%)	0 (0%)	0 (0%)	0 (0%)	1 (13%)
15	4 (100%)	294 (99%)	270 (100%)	9 (100%)	3 (100%)	18 (100%)	60 (100%)	7 (88%)
Regional trauma network grade (A–C)								
C	–	20 (100%)	25 (100%)	1 (100%)	–	1 (100%)	7 (100%)	1 (100%)
Missing	4	276	246	8	3	17	53	7

**Table 2:** Injury assessment, imaging, and management by sex and age group

Sex	Female				Male			
Age (years)	< 5 *N* = 4^1^	[5; 18) *N* = 296^1^	[18; 60] *N* = 271^1^	> 60 *N* = 9^1^	< 5 *N* = 3^1^	[5; 18) *N* = 18^1^	[18; 60] *N* = 60^1^	> 60 *N* = 8^1^
Abnormal examination								
Chest	0 (0%)	10 (3.4%)	8 (3.0%)	1 (11%)	0 (0%)	1 (5.6%)	7 (12%)	3 (38%)
Abdomen	0 (0%)	10 (3.4%)	11 (4.1%)	0 (0%)	0 (0%)	1 (5.6%)	5 (8.3%)	0 (0%)
Lower limb	1 (25%)	74 (25%)	80 (30%)	0 (0%)	0 (0%)	1 (5.6%)	20 (33%)	3 (38%)
Missing	0	1	0	0				
Upper limb	3 (75%)	120 (41%)	66 (24%)	6 (67%)	2 (67%)	7 (39%)	18 (30%)	1 (13%)
Spine	0 (0%)	119 (40%)	134 (49%)	4 (44%)	1 (33%)	6 (33%)	19 (32%)	3 (38%)
Neurological	0 (0%)	37 (13%)	53 (20%)	2 (22%)	0 (0%)	3 (17%)	9 (15%)	2 (25%)
Skin	0 (0%)	16 (5.4%)	19 (7.0%)	2 (22%)	0 (0%)	0 (0%)	5 (8.3%)	0 (0%)
Imaging								
MRI	0 (0%)	1 (0.3%)	4 (1.5%)	0 (0%)	0 (0%)	0 (0%)	3 (5.0%)	0 (0%)
Abnormal	0 (0%)	0 (0%)	4 (1.5%)	0 (0%)	0 (0%)	0 (0%)	2 (3.3%)	0 (0%)
Not performed	4 (100%)	295 (100%)	267 (99%)	9 (100%)	3 (100%)	18 (100%)	57 (95%)	8 (100%)
Normal	0 (0%)	1 (0.3%)	0 (0%)	0 (0%)	0 (0%)	0 (0%)	1 (1.7%)	0 (0%)
CT	0 (0%)	79 (27%)	114 (42%)	5 (56%)	0 (0%)	4 (22%)	30 (50%)	5 (63%)
Abnormal	0 (0%)	29 (9.8%)	47 (17%)	4 (44%)	0 (0%)	1 (5.6%)	19 (32%)	3 (38%)
Not performed	4 (100%)	217 (73%)	156 (58%)	4 (44%)	3 (100%)	14 (78%)	30 (50%)	3 (38%)
Normal	0 (0%)	50 (17%)	68 (25%)	1 (11%)	0 (0%)	3 (17%)	11 (18%)	2 (25%)
Radiography	4 (100%)	222 (75%)	180 (66%)	6 (67%)	2 (67%)	10 (56%)	36 (60%)	6 (75%)
Abnormal	2 (50%)	87 (29%)	54 (20%)	3 (33%)	2 (67%)	3 (17%)	15 (25%)	4 (50%)
Not performed	0 (0%)	75 (25%)	92 (34%)	3 (33%)	1 (33%)	8 (44%)	23 (38%)	2 (25%)
Normal	2 (50%)	134 (45%)	125 (46%)	3 (33%)	0 (0%)	7 (39%)	21 (35%)	2 (25%)
Whole-body CT	0 (0%)	12 (4.1%)	16 (5.9%)	0 (0%)	0 (0%)	0 (0%)	9 (15%)	0 (0%)
Abnormal	0 (0%)	3 (1.0%)	10 (3.7%)	0 (0%)	0 (0%)	0 (0%)	5 (8.3%)	0 (0%)
Not performed	4 (100%)	285 (96%)	255 (94%)	9 (100%)	3 (100%)	18 (100%)	51 (85%)	8 (100%)
Normal	0 (0%)	8 (2.7%)	6 (2.2%)	0 (0%)	0 (0%)	0 (0%)	4 (6.7%)	0 (0%)
Ultrasonography	0 (0%)	16 (5.4%)	8 (3.0%)	0 (0%)	0 (0%)	0 (0%)	2 (3.3%)	0 (0%)
Abnormal	0 (0%)	1 (0.3%)	2 (0.7%)	0 (0%)	0 (0%)	0 (0%)	1 (1.7%)	0 (0%)
Not performed	4 (100%)	280 (95%)	263 (97%)	9 (100%)	3 (100%)	18 (100%)	58 (97%)	8 (100%)
Normal	0 (0%)	15 (5.1%)	6 (2.2%)	0 (0%)	0 (0%)	0 (0%)	1 (1.7%)	0 (0%)
Incidental findings	0 (0%)	2 (0.7%)	4 (1.5%)	0 (0%)	0 (0%)	0 (0%)	2 (3.3%)	0 (0%)
Radiation dose (Gy)	–	789 (528, 1,293)	942 (584, 1,532)	1,067 (606, 2,145)	–	938 (471, 1,383)	1,403 (765, 2,034)	993 (941, 1,261)
Missing	4	232	182	4	3	14	39	3
Overall imaging results								
Abnormal	2 (50%)	108 (36%)	81 (30%)	6 (67%)	2 (67%)	4 (22%)	27 (45%)	4 (50%)
Not performed	0 (0%)	22 (7.4%)	15 (5.5%)	0 (0%)	1 (33%)	4 (22%)	2 (3.3%)	0 (0%)
Normal	2 (50%)	166 (56%)	175 (65%)	3 (33%)	0 (0%)	10 (56%)	31 (52%)	4 (50%)
Disposition								
Ward admission	1 (25%)	52 (18%)	40 (15%)	6 (67%)	0 (0%)	3 (17%)	14 (23%)	5 (63%)
Discharged home	3 (75%)	237 (80%)	225 (83%)	3 (33%)	3 (100%)	15 (83%)	44 (73%)	3 (38%)
Intensive care	0 (0%)	0 (0%)	1 (0.4%)	0 (0%)	0 (0%)	0 (0%)	0 (0%)	0 (0%)
Interfacility transfer	0 (0%)	7 (2.4%)	5 (1.8%)	0 (0%)	0 (0%)	0 (0%)	2 (3.3%)	0 (0%)
Surgery during index care	1 (25%)	26 (8.8%)	14 (5.2%)	1 (11%)	0 (0%)	2 (11%)	11 (18%)	2 (25%)
Vertebral cementoplasty	0 (0%)	0 (0%)	2 (17%)	0 (0%)	–	0 (0%)	2 (25%)	0 (0%)
Kyphoplasty	0 (0%)	0 (0%)	0 (0%)	0 (0%)	–	0 (0%)	1 (13%)	0 (0%)
Osteosynthesis	1 (100%)	16 (76%)	9 (75%)	1 (100%)	–	1 (100%)	5 (63%)	2 (100%)
Reduction under general anesthesia	0 (0%)	5 (24%)	0 (0%)	0 (0%)	–	0 (0%)	0 (0%)	0 (0%)
Not concerned	3	275	259	8	3	17	52	6
Length of stay (days)								
Median (Q1, Q3)	1.00 (1.00, 1.00)	2.00 (1.00, 3.00)	2.00 (1.00, 4.00)	1.00 (1.00, 2.00)	–	1.00 (1.00, 1.00)	3.00 (2.00, 5.00)	2.00 (1.00, 3.00)
Not hospitalized	3	245	232	3	3	16	46	5
Protective equipment								
Helmet	1 (100%)	148 (97%)	127 (99%)	3 (60%)	1 (100%)	9 (100%)	28 (97%)	4 (100%)
Missing	3	143	143	4	2	9	31	4
Back protector	0 (0%)	49 (51%)	24 (33%)	1 (33%)	0 (0%)	1 (25%)	1 (5.0%)	2 (67%)
Missing	4	200	198	6	2	14	40	5
Injury Severity Score	2.50 (1.00, 4.00)	3.00 (1.00, 4.00)	2.00 (1.00, 4.00)	6.00 (4.00, 14.00)	4.0 (2.0, 4.0)	2.0 (1.0, 4.0)	4.0 (2.0, 5.0)	4.0 (2.0, 6.5)

*1n (%)* Median (Q1, Q3), *CT* computed tomography, *MRI* magnetic resonance imaging, *ED* emergency department

**Table 3:** Comparison of imaging, disposition, and injury severity by age group

**Age (years)**	**< 5** *N* = 7^1^	**[5; 18)** *N* = 314^1^	**[18; 60]** *N* = 331^1^	**> 60** *N* = 17^1^	***p*-value**^2^
**Overall imaging result**					0.066
Abnormal	4 (67%)	112 (39%)	108 (34%)	10 (59%)	
Normal	2 (33%)	176 (61%)	206 (66%)	7 (41%)	
Not performed	1	26	17	0	
**Disposition**					**< 0.001**
Admission	1 (14%)	62 (20%)	62 (19%)	11 (65%)	
Discharged home	6 (86%)	252 (80%)	269 (81%)	6 (35%)	
**Injury Severity Score**	4.00 (1.00, 4.00)	3.00 (1.00, 4.00)	2.00 (1.00, 4.00)	5.00 (3.00, 9.00)	**0.025**

^1^n (%); Median (Q1, Q3)

^2^Fisher's exact test; Kruskal–Wallis rank sum test

**Table 4:** Comparison of imaging, disposition, and injury severity by sex

**Age (years)**	**Female** *N* = 580^1^	**Male** *N* = 89^1^	***p*-value**^2^
**Overall imaging result**			0.12
Abnormal	197 (36%)	37 (45%)	
Normal	346 (64%)	45 (55%)	
Not performed	37	7	
**Disposition**			0.095
Admission	112 (19%)	24 (27%)	
Discharged home	468 (81%)	65 (73%)	
**Injury Severity Score**	3.00 (1.00, 4.00)	4.00 (1.00, 5.00)	0.078

^*1*^*n (%)* Median (Q1, Q3)

^2^Pearson's chi-squared test; Wilcoxon rank-sum test

### Incidence

Using the 2021 population data from the Institut national de la statistique et des études économiques, the cumulative incidence across all catchments areas was 327.6 per 100,000 inhabitants between May 2021 and October 2024, corresponding to an average annual incidence of 93.6 per 100,000 person-years. By catchment area, the incidence rates were as follows: Avranches–Granville–Saint-Hilaire, 29.0/100,000/year; Cherbourg, 90.2/100,000/year; and Saint-Lô–Coutances, 166.7/100,000/year. A highly significant heterogeneity in incidence rates was observed across the three catchments (likelihood ratio test, χ^2^ with 2 degrees of freedom, *p* < 2.2 × 10⁻^1^⁶), indicating that incidence rates were not homogeneous between areas. When standardized for Fédération Française d’Équitation licensees (*N* = 7,234 in 2024), the cumulative incidence was 9,251 per 100,000 licensees, corresponding to an annual incidence of 2,643 per 100,000 licensees.

### Protective equipment

Among patients with documented data, helmet use was reported in 97% (N = 321 with documentation), whereas back protection was recorded in 39% (*N* = 78 with documentation).

### Injury profile

**Fig. 3:**
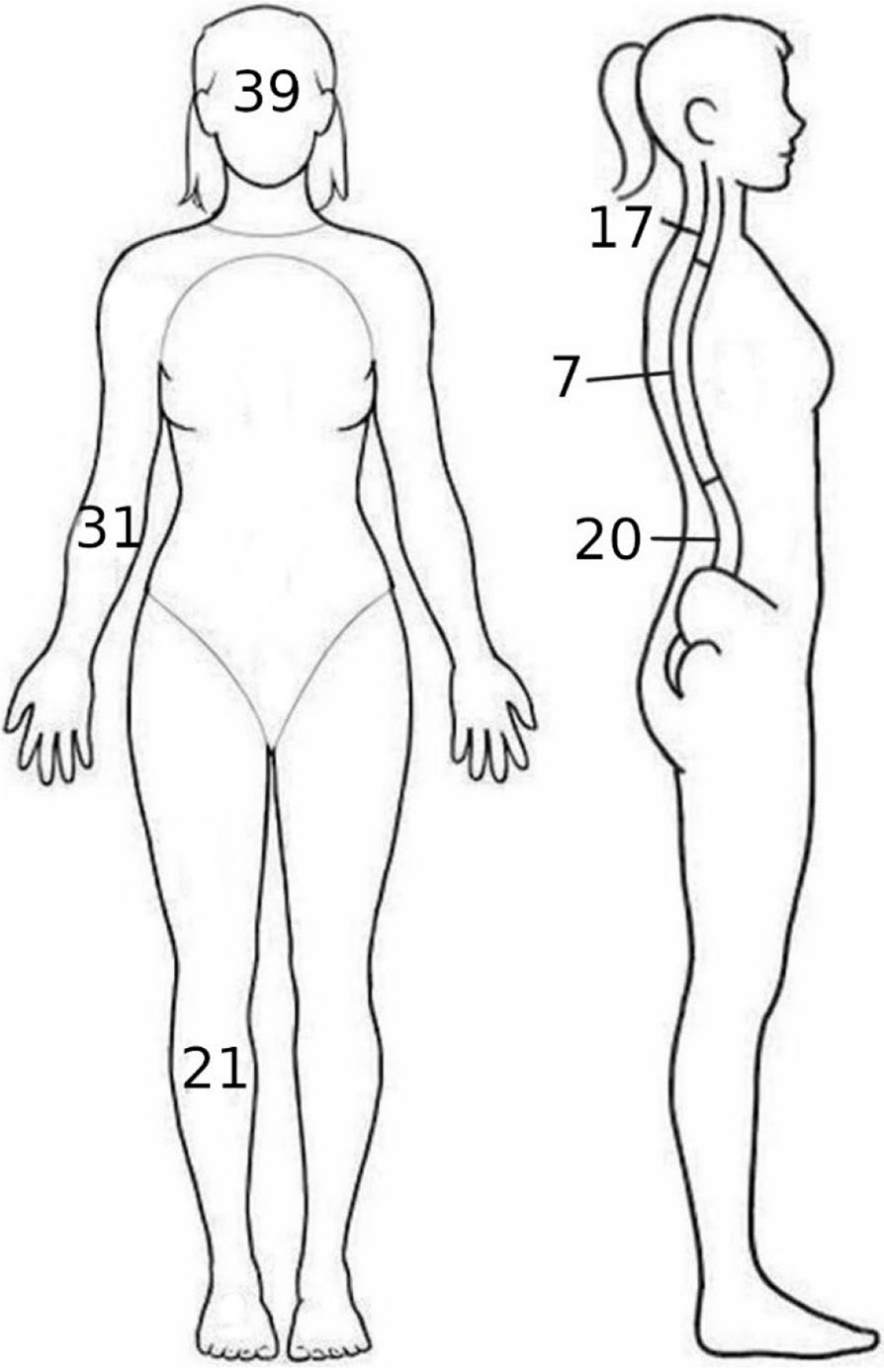
Injury distribution by body region (% of subjects). Note: Values are presented as percentages. Percentages do not sum to 100% because subjects could sustain multiple injuries

The most frequently injured body regions (not mutually exclusive, as patients could sustain injuries to multiple regions) were the spine (*N* = 288, 43%), head (*N* = 258, 39%), upper limbs (*N* = 206, 33%), lower limbs (*N* = 179, 27%), and pelvis (*N* = 115, 17%) (Fig. 3). Head trauma included five intracranial bleedings and three skull fractures; initial loss of consciousness occurred in 48 patients (19% of head injuries). Spinal injuries were predominantly contusions and stable fractures across cervical, thoracic, and lumbar segments. Limb injuries were primarily contusions and fractures, with upper limb fractures (*N* = 74) being the most common fracture type overall. Severe injuries (AIS ≥ 3) were infrequent, occurring in the head (*N* = 7, 1.0%), upper limbs (*N* = 5, 0.7%), thorax (*N* = 9, 1.3%), and lower limbs (*N* = 3, 0.4%). The overall median ISS was 3 (IQR, 1–4), indicating that most injuries were minor. However, age-stratified analysis revealed significant differences in injury severity: patients aged > 60 years had the highest median ISS (5.0; IQR, 3.0–9.0), compared to 4.0 (IQR, 1.0–4.0) in the < 5 year group, 3.0 (IQR, 1.0–4.0) in the 5–18 year group, and 2.0 (IQR, 1.0–4.0) in the 19–60 year group (*p* = 0.025, Kruskal–Wallis test). Sex-stratified analysis showed a trend toward higher ISS in men (median 4.0, IQR 1.0–5.0) than in women (median 3.0, IQR 1.0–4.0; *p* = 0.078, Wilcoxon rank-sum test). The complete injury distribution by anatomical region, type, and AIS severity is detailed in Table 2 and Supplementary Tables S1–S3.

### Imaging utilization and findings

At least one imaging test was performed in 93% of visits (*N* = 625). Radiography was the most frequently used modality (*N* = 466) with 37% showing abnormalities, followed by CT (*N* = 237, 43% abnormal) and whole-body CT (*N* = 37, 49% abnormal). CT utilization increased with age: 26% in patients aged 5–18 years, 44% in the 19–60 year group, and 59% in patients > 60 years (Table 2). Abnormal imaging findings were more frequent in men (45%) than in women (36%; *p* = 0.12).

### Management and early outcomes

After ED management, 533 patients (80%) were discharged home, 121 (18%) required hospitalization (median stay 2 days; IQR, 1–4), one was admitted to the Intensive Care Unit, and 14 (2.1%) were transferred for specialized care. Fifty-seven patients (8.5%) underwent surgery, primarily osteosynthesis (*N* = 35, 71%). Admission rates were significantly higher in patients aged > 60 years (65%) than in younger groups (*p* < 0.001), and showed a trend toward being higher in men (27% vs. 19%; *p* = 0.095). Detailed management data are presented in Tables 2, 3 and 4.

## Discussion

​In this multicenter cohort study from a French department with dense equestrian activity, 669 ED presentations after falls from a horse were identified over 3.5 years (May 2021–October 2024). Horse riding-related falls accounted for approximately 0.15% of all ED visits across participating centers, with substantial geographic variation across catchments. The injuries predominantly involved the head and spine; however, severe neurocranial complications were rare, and most patients were discharged after a brief evaluation. The high rate of helmet use coincided with a low frequency of severe head injuries, suggesting a protective role; however, without a comparator group of unhelmeted riders, causal inference cannot be made. Age-stratified analysis revealed that patients aged > 60 years had significantly higher injury severity (median ISS, 5.0 vs. 2.0–3.0 in younger groups; *p* = 0.025) and hospitalization rates (65% vs. 19%–20%; *p* < 0.001). Male riders demonstrated a trend toward greater injury severity than female riders (median ISS 4.0 vs. 3.0; *p* = 0.078).

Our study results align with international trends in horse riding-related injuries while providing a specific regional context through exhaustive data collection. As described by Hoffmann et al. and Meredith et al. [21, 22], women predominance among injured riders reflects rider demographics in Western countries. Women constituted 87% of our cohort, consistent with licensed rider demographics in France where 84% of equestrian federation members are women [23]. This sex distribution has implications for injury prevention, as male riders tended to sustain more severe injuries (median ISS, 4.0; IQR 1.0–5.0 vs. 3.0, IQR 1.0–4.0; *p* = 0.078) and had higher hospitalization rates (27% vs. 19%; *p* = 0.095), suggesting that male riders may engage in higher-risk activities or experience higher-energy trauma. The high proportion of young women—particularly in the 5–18 year age group (*N* = 314, 47% of the cohort)—raises concerns regarding long-term musculoskeletal consequences and the need for age- and sex-tailored prevention strategies [23]. The median age of 18 years (IQR 13–29) was substantially lower than that reported in many specialized center-based series, which typically report mean ages ranging from 30 to 40 years [24, 25]. This difference stems from the inclusion of minors in our data collection, which provided a comprehensive view of equestrian accidents.

To contextualize the burden of horse riding-related injuries, the 18% hospitalization rate and 8.5% surgery rate in our cohort substantially exceed those typically reported for cycling (approximately 4% hospitalization) [26] and skiing (approximately 4.5%) [27] in EDs. These findings are consistent with previous equestrian cohort studies reporting hospitalization rates of 10%–25% [28], confirming the comparatively high severity of equestrian falls among recreational activities. The median ISS of 3 in our cohort reflects predominantly minor injuries; however, the predominance of spinal injuries (43% of patients) substantially exceeds that observed in most other recreational activities, supporting the classification of horse riding as a high-risk activity for axial skeletal injury. The high incidence in the Saint-Lô–Coutances catchment area (166.7/100,000 inhabitants per year) illustrates the impact of dense equestrian club concentration, a phenomenon noted in regional sports studies. The anatomical distribution confirmed previous findings that the head, spine, and upper limbs were the most affected, as reported by Ballet al. and Kłusek et al. [3, 6]. The incidence of severe head injuries remains low, likely due to high helmet use, confirming Bier et al.’s findings on helmet effectiveness [29]. Spinal injuries, present in 43% of visits, are comparable to those in hospital cohorts but show mostly stable fractures and contusions [6, 30, 31]. Our data showed fewer chest and pelvic injuries than those in international studies [25], likely because leisure activities, which predominates among amateur riders in rural Manche, generate lower kinetic energy (e.g., lower speed and impact than high-speed competitions). The lower incidence of serious abdominal injuries than that reported by Weber et al. confirms their rarity without crushing mechanisms [16]. Imaging was performed in nearly all ED visits, with CT use increasing in older patients, reflecting careful evaluation for occult fractures. This systematic approach likely facilitated the detection of clinically relevant injuries, including minor trauma that may have been missed in more selective series.

### Strengths and limitations

This study has several strengths. Its comprehensive scope is a major asset; with 669 riders over 3.5 years, it represents one of the largest recent cohorts studying horse riding-related fall injuries. The multicenter nature enhances data representativeness by including various practices while limiting single-center recruitment bias. Standardized data collection based on a harmonized form and systematic computerized extraction of imaging and injury scores ensure methodological consistency. This study covers the post-pandemic period when horse riding increased, providing a contemporary view of equestrian traumatology.

However, this study has some limitations. The multicenter approach improves representativeness within the Manche Department, a region with high equestrian activity, reflecting a specific rural context; nevertheless, this limits generalizability to regions with lower levels of equine involvement. Retrospective data collection depends on the quality and completeness of medical records. Key contextual variables, such as rider experience, activity type, and fall mechanisms, were unavailable even in SMUR records for severe cases, restricting etiological insights and prevention recommendations. The use of protective equipment was not systematically recorded, limiting the risk factor analysis. The absence of a comparison group restricts etiological conclusions, particularly regarding helmet efficacy, highlighting the need for prospective controlled studies. Although severity assessment uses standardized scores, it depends on the completeness of the diagnosis. Minor injuries may have been underestimated, whereas systematic imaging in some centers may have detected clinically insignificant injuries, creating a measurement bias. The lack of uniform imaging protocols likely contributes to detection variations. Additionally, focusing on acute care without longitudinal follow-up prevented the assessment of functional progress, sequelae, or activity resumption, thereby limiting conclusions regarding the overall impact of trauma. Finally, the identification of seven patients directly transferred from prehospital care to an external trauma center indicates that our ED-based inclusion may have missed a small number of severe cases, potentially leading to a slight underestimation of incidence (approximately 1% additional cases) and an underrepresentation of high-severity injuries (e.g., those requiring immediate specialized intervention). Missed cases may also include rare isolated on-site fatalities without SMUR involvement, although the French SAMU/SMUR system ensures most prehospital deaths are documented and potentially captured. Minor injuries treated in primary care or self-managed are not represented, limiting assessment of less severe outcomes and overall incidence. This underscores the need for future studies to integrally incorporate prehospital, primary care, and interfacility transfer data.

### Future directions

The results of this study open several avenues for future studies and prevention. A prospective study at a regional or national level focusing on horse falls can overcome the limitations of retrospective data collection. It would gather data that are currently lacking, such as rider experience, activity type, fall circumstances, and impact nature. Analysis of these variables would help identify at-risk groups and enable the development of preventive strategies.

Further studies are required to evaluate the effectiveness of modern protective equipment. Although the protective role of helmets has been documented [9], precise quantification of concussion and skull fracture reduction is required in French populations, particularly in the lateral areas. The recently introduced helmets with temporal protection may better protect the fragile area of the skull housing the middle cerebral artery. Epidemiological studies can be used to assess whether these models reduce lateral head injuries.

Future studies can validate a clinical decision rule incorporating helmet/back protector use, fall mechanism, initial GCS score, and the absence of focal neurological deficits, aiming for a high NPV to reduce unnecessary CT scans in low-risk patients, similar to existing trauma algorithms. Additionally, the observed variations between areas support targeted prevention and outreach efforts, such as mandatory equipment audits in high-incidence equestrian clubs and community education programs focused on fall risk reduction.

Protective vests, particularly airbag models with neck protection, require biomechanical and clinical studies to assess their effectiveness in preventing spinal and thoracic fractures. Although these instantaneously inflating vests represent innovation, data remain insufficient to prove their effectiveness. Some analyses of eventing competitions suggest that airbags may not reduce serious injury risk but can increase the risk of severe injury in certain cases [10]. The identification of specific injury mechanisms justifies the development of targeted devices, such as improved safety stirrups and supports. Prevention requires training through educational programs to control falls, risk management incorporation, and the recognition of dangerous horse behaviors. Regarding prevention, several promising measures have been implemented internationally with demonstrated efficacy. In Sweden, public health campaigns emphasizing helmet use and rider education—including attention to predictors such as unmounted injuries (e.g., kicks)—have reduced equestrian-related, achieving a 20%–30% decline in injuries in monitored cohorts [28]. These interventions highlight the value of targeted, evidence-based prevention strategies without overgeneralization. These initiatives, along with epidemiological monitoring, would create an integrated approach combining protection, innovation, and education.

## Conclusion

Horse riding-related falls represent a common cause of ED visits in high-density equestrian regions such as Manche, with substantial geographic variation. Although most injuries are minor, the high prevalence of head and spine trauma underscores the importance of protective equipment and targeted prevention strategies. Implementing standardized imaging and discharge pathways can optimize resource utilization while ensuring the identification of clinically significant injuries.

## Supplementary Information

Supplementary Material 1.

Supplementary Material 2.

Supplementary Material 3.

## Data Availability

Data are available upon reasonable request.

## Abbreviations

ED: Emergency Department
IQR: Interquartile Range
SD: Standard Deviation
SAMU: Service d’Aide Médicale Urgente/Emergency Medical Assistance Service
SMUR: Service Mobile d’Urgence et de Réanimation/Emergency and Resuscitation Mobile Service
STROBE: Strengthening the Reporting of Observational Studies in Epidemiology

## References

[1] FEI. FEI Annual Report 2024. https://inside.fei.org/uploads/FEI%20Annual%20Report%202024.pdf. Accessed 29 Jun 2025.

[2] Foreman MH, Engsberg JR, Foreman JH. Point mass impulse-momentum model of the equine rotational fall. Comp Exerc Physiol. 2019;15:157–66.

[3] Ball CG, Ball JE, Kirkpatrick AW, Mulloy RH. Equestrian injuries: incidence, injury patterns, and risk factors for 10 years of major traumatic injuries. Am J Surg. 2007;193:636–40. 17434372 10.1016/j.amjsurg.2007.01.016

[4] Mutore K, Lim J, Fofana D, Torres-Reveron A, Skubic JJ. Hearing hoofbeats? Think head and neck trauma: a 10-year NTDB analysis of equestrian-related trauma in the USA. Trauma Surg Acute Care Open. 2021;6:e000728. 34595353 10.1136/tsaco-2021-000728 PMC8442081

[5] Ingemarson H, Grevsten S, Thorén L. Lethal horse-riding injuries. J Trauma. 1989;29:25–30. 2911099 10.1097/00005373-198901000-00005

[6] Kłusek M, Chawrylak K, Kuszneruk J, Kubas M, Krzemińska K. Epidemiology and evaluation of spine injuries in equestrian sports. Qual Sport. 2024;17:53095.

[7] Abu-Zidan FM, Rao S. Factors affecting the severity of horse-related injuries. Injury. 2003;34:897–900. 14636730 10.1016/s0020-1383(03)00054-8

[8] Laurent R, Uhring J, Bentahar M, Constantinou B, De Billy B, Langlais J. Epidemiology of pediatric equestrian injuries. Arch Pediatr. 2012;19:1053–7. 22981477 10.1016/j.arcped.2012.07.010

[9] Bier G, Bongers MN, Othman A, Hempel JM, Vieth V, Heindel W, et al. Impact of helmet use in equestrian-related traumatic brain injury: a matched-pairs analysis. Br J Neurosurg. 2018;32:37–43. 29205071 10.1080/02688697.2017.1409874

[10] Nylund LE, Sinclair PJ, Hitchens PL, Cobley S. Do riders who wear an air jacket in equestrian eventing have reduced injury risk in falls? A retrospective data analysis. J Sci Med Sport. 2019;22:1010–3. 31160233 10.1016/j.jsams.2019.05.012

[11] Drapeau C. Chiffre sur les activités équestre. IFCE; 2022 p. 5. https://equipedia.ifce.fr/economie-et-filiere/economie/chiffres-cles-de-la-filiere/les-chiffres-sur-les-activites-equestres?tx_web2pdf_pi1%5Baction%5D=&tx_web2pdf_pi1%5Bargument%5D=printPage&tx_web2pdf_pi1%5Bcontroller%5D=Pdf&cHash=3cafd93916b31f06fe43be6e54d405a4. Accessed 29 Jun 2025.

[12] Thélot B. Épidémiologie des accidents traumatiques en pratique sportive en France. Bull Épidémiol Hebd. 2015;12:200–5.

[13] Boiron A, Barazer C, Clement C, Sahli Vivicorsi S, Bellamy L, Le Toux G, et al. Craniofacial trauma of equestrian origin. J Craniofac Surg. 2024;35:1607–11. 38810245 10.1097/SCS.0000000000010126 PMC11346711

[14] IFCE. Nombres de cavaliers licenciés. IFCE; 2024. https://statscartes.ifce.fr/utilisations/equitation/nombre-de-licencis_d32. Accessed 29 Jun 2025.

[15] Nguyen HS, Lew S. Equestrian-related traumatic brain injury in the pediatric population. Pediatr Neurosurg. 2016;51:279–83. 27322378 10.1159/000446402

[16] Weber CD, Nguyen AR, Lefering R, Hofman M, Hildebrand F, Pape HC. Blunt injuries related to equestrian sports: results from an international prospective trauma database analysis. Int Orthop. 2017;41:2105–12. 28801837 10.1007/s00264-017-3592-1

[17] Havlik HS. Equestrian sport-related injuries: a review of current literature. Curr Sports Med Rep. 2010;9:299. 20827097 10.1249/JSR.0b013e3181f32056

[18] Insee. Dossier complet − Département de la Manche (50). https://www.insee.fr/fr/statistiques/2011101?geo=DEP-50. Accessed 23 Jan 2026.

[19] IFCE. IFCE Leaflet: Key Figures 2024. https://equipedia.ifce.fr/bibliotheque/6.Statistiques/6.1.Ecus-depliant/IFCE-Leaflet-key-figures-2024.pdf. Accessed 23 Jan 2026.

[20] Traumabase. Codage ISS Final. https://www.traumabase.eu/document/codissfi/Codage%20ISS%20final.pdf. Accessed 11 Feb 2026.

[21] Meredith L, Thomson R, Ekman R, Kovaceva J, Ekbrand H, Bálint A. Equestrian-related injuries, predictors of fatalities, and the impact on the public health system in Sweden. Public Health. 2019;168:67–75. 30690221 10.1016/j.puhe.2018.11.023

[22] Hoffmann MF, Bernstorff M, Kreitz N, Roetman B, Schildhauer TA, Wenning KE. Horse-related injury patterns: a single center report. J Orthop Surg Res. 2023;18:83. 36732813 10.1186/s13018-023-03549-3 PMC9893574

[23] Franzén Lindgren E, Hammarqvist F, Ahl Hulme R. Horse-riding hazards: an observational cohort study mapping equestrian related injuries at a Scandinavian trauma centre. BMC Sports Sci Med Rehabil. 2023;15:46. 36978116 10.1186/s13102-023-00646-y PMC10045660

[24] Acton AS, Gaw CE, Chounthirath T, Smith GA. Nonfatal horse-related injuries treated in emergency departments in the United States, 1990–2017. Am J Emerg Med. 2020;38:1062–8. 31402233 10.1016/j.ajem.2019.158366

[25] Adler CR, Hopp A, Hrelic D, Patrie JT, Fox MG. Retrospective analysis of equestrian-related injuries presenting to a level 1 trauma center. Emerg Radiol. 2019;26:639–45. 31435897 10.1007/s10140-019-01718-8

[26] Sanford T, McCulloch CE, Callcut RA, Carroll PR, Breyer BN. Bicycle trauma injuries and hospital admissions in the United States, 1998-2013. JAMA. 2015;314:947–9. 26325564 10.1001/jama.2015.8295 PMC4896174

[27] Yendluri A, Hrabarchuk EI, Obana KK, Namiri NK, Plancher KD, Trofa DP, et al. Skiing injuries in the US pediatric population: an analysis of national injury trends and mechanisms between 2012 and 2022. Orthop J Sports Med. 2024;12:23259671241255704. 38911123 10.1177/23259671241255704 PMC11193339

[28] Franzén Lindgren E, Hammarqvist F, Ahl Hulme R. Horse-riding hazards: an observational cohort study mapping equestrian related injuries at a Scandinavian trauma centre. BMC Sports Sci Med Rehabil. 2023;15:46. 36978116 10.1186/s13102-023-00646-y PMC10045660

[29] Bier G, Bongers MN, Othman A, Hempel JM, Vieth V, Heindel W, et al. Impact of helmet use in equestrian-related traumatic brain injury: a matched-pairs analysis. Br J Neurosurg. 2018;32:37–43. 29205071 10.1080/02688697.2017.1409874

[30] Schicho A, Einwag D, Eickhoff A, Richter PH, Riepl C. Impact of spinal fractures in horseback riding. Sportverletz Sportschaden. 2015;29:231–5. 26574887 10.1055/s-0041-106944

[31] Lin CY, Wright J, Bushnik T, Shem K. Traumatic spinal cord injuries in horseback riding: a 35-year review. Am J Sports Med. 2011;39:2441–6. 21856930 10.1177/0363546511419280

