# Supplementary material for: Characteristics of horse riding-related falls in patients presenting to emergency departments in manche department, france: a multicenter retrospective analysis
Source: Scand J Trauma Resusc Emerg Med. 2026 Mar 10;34:76. doi: 10.1186/s13049-026-01593-6 (PMC13088590; doi:10.1186/s13049-026-01593-6)
Supplement: Supplementary file 1 — Supplementary Material 1. [file 13049_2026_1593_MOESM1_ESM.docx]

Supplementary Table 1 (S1) : Detailed limb examination findings by sex and age group

|  | **Female** | | | | **Male** | | | | |
| --- | --- | --- | --- | --- | --- | --- | --- | --- | --- |
| **Age (years)** | **< 5**  N = 4^1^ | **[5 ; 18)**  N = 296^1^ | **[18 ; 60]**  N = 271^1^ | **> 60**  N = 9^1^ | **< 5**  N = 3^1^ | **[5 ; 18)**  N = 18^1^ | **[18 ; 60]**  N = 60^1^ | **> 60**  N = 8^1^ |  |
| **Upper limb severity (0-3)** |  |  |  |  |  |  |  |  |  |
| 0 | 1 (25%) | 184 (62%) | 209 (77%) | 3 (33%) | 2 (67%) | 11 (61%) | 46 (77%) | 7 (88%) |  |
| 1 | 1 (25%) | 59 (20%) | 40 (15%) | 4 (44%) | 0 (0%) | 4 (22%) | 9 (15%) | 1 (13%) |  |
| 2 | 2 (50%) | 50 (17%) | 20 (7.4%) | 2 (22%) | 1 (33%) | 3 (17%) | 5 (8.3%) | 0 (0%) |  |
| 3 | 0 (0%) | 3 (1.0%) | 2 (0.7%) | 0 (0%) | 0 (0%) | 0 (0%) | 0 (0%) | 0 (0%) |  |
| **Upper limb injuries** |  |  |  |  |  |  |  |  |  |
| **Fracture** | 2 (50%) | 49 (17%) | 14 (5.2%) | 2 (25%) | 1 (33%) | 2 (11%) | 4 (6.7%) | 0 (0%) |  |
| Missing | 0 | 0 | 0 | 1 | 0 | 0 | 0 | 0 |  |
| **Contusion** | 1 (25%) | 51 (17%) | 37 (14%) | 4 (44%) | 0 (0%) | 4 (22%) | 6 (10%) | 0 (0%) |  |
| **Laceration** | 0 (0%) | 3 (1.0%) | 5 (1.8%) | 1 (11%) | 0 (0%) | 0 (0%) | 1 (1.7%) | 1 (13%) |  |
| **Sprain** | 0 (0%) | 6 (2.0%) | 5 (1.8%) | 0 (0%) | 0 (0%) | 1 (5.6%) | 3 (5.0%) | 0 (0%) |  |
| **Dislocation** | 0 (0%) | 9 (3.0%) | 7 (2.6%) | 0 (0%) | 0 (0%) | 1 (5.6%) | 2 (3.3%) | 0 (0%) |  |
| **Upper limb fracture type** |  |  |  |  |  |  |  |  |  |
| Simple | 1 (100%) | 19 (63%) | 7 (64%) | 0 (0%) | 0 (0%) | 2 (100%) | 1 (50%) | – |  |
| Articular | 0 (0%) | 4 (13%) | 2 (18%) | 1 (100%) | 1 (100%) | 0 (0%) | 0 (0%) | – |  |
| Comminuted | 0 (0%) | 3 (10%) | 0 (0%) | 0 (0%) | 0 (0%) | 0 (0%) | 0 (0%) | – |  |
| Displaced | 0 (0%) | 4 (13%) | 2 (18%) | 0 (0%) | 0 (0%) | 0 (0%) | 1 (50%) | – |  |
| Missing | 3 | 266 | 260 | 8 | 2 | 16 | 58 | 8 |  |
| **Lower limb severity (0-3)** |  |  |  |  |  |  |  |  |  |
| 0 | 3 (75%) | 238 (80%) | 207 (76%) | 9 (100%) | 3 (100%) | 17 (94%) | 48 (80%) | 6 (75%) |  |
| 1 | 1 (25%) | 42 (14%) | 54 (20%) | 0 (0%) | 0 (0%) | 1 (5.6%) | 8 (13%) | 0 (0%) |  |
| 2 | 0 (0%) | 14 (4.7%) | 9 (3.3%) | 0 (0%) | 0 (0%) | 0 (0%) | 4 (6.7%) | 2 (25%) |  |
| 3 | 0 (0%) | 2 (0.7%) | 1 (0.4%) | 0 (0%) | 0 (0%) | 0 (0%) | 0 (0%) | 0 (0%) |  |
| **Lower limb injuries** |  |  |  |  |  |  |  |  |  |
| Fracture | 0 (0%) | 11 (3.7%) | 6 (2.2%) | 0 (0%) | 0 (0%) | 0 (0%) | 4 (6.7%) | 2 (25%) |  |
| Contusion | 1 (25%) | 39 (13%) | 44 (16%) | 0 (0%) | 0 (0%) | 1 (5.6%) | 6 (10%) | 0 (0%) |  |
| Skin laceration | 0 (0%) | 0 (0%) | 0 (0%) | 0 (0%) | 0 (0%) | 0 (0%) | 0 (0%) | 0 (0%) |  |
| Sprain | 0 (0%) | 11 (3.7%) | 13 (4.8%) | 0 (0%) | 0 (0%) | 0 (0%) | 2 (3.3%) | 0 (0%) |  |
| Dislocation | 0 (0%) | 3 (1.0%) | 1 (0.4%) | 0 (0%) | 0 (0%) | 0 (0%) | 1 (1.7%) | 0 (0%) |  |
| **Type of lower-limb fracture** |  |  |  |  |  |  |  |  |  |
| Simple | – | 3 (60%) | 2 (50%) | – | – | – | 0 (0%) | 0 (0%) |  |
| Articular | – | 0 (0%) | 1 (25%) | – | – | – | 1 (25%) | 1 (50%) |  |
| Comminuted | – | 0 (0%) | 1 (25%) | – | – | – | 1 (25%) | 1 (50%) |  |
| Displaced | – | 2 (40%) | 0 (0%) | – | – | – | 2 (50%) | 0 (0%) |  |
| Missing | 4 | 291 | 267 | 9 | 3 | 18 | 56 | 6 |  |
| ^1^n (%) | | | | | | | | |  |
