# Supplementary material for: Characteristics of horse riding-related falls in patients presenting to emergency departments in manche department, france: a multicenter retrospective analysis
Source: Scand J Trauma Resusc Emerg Med. 2026 Mar 10;34:76. doi: 10.1186/s13049-026-01593-6 (PMC13088590; doi:10.1186/s13049-026-01593-6)
Supplement: Supplementary file 2 — Supplementary Material 2. [file 13049_2026_1593_MOESM2_ESM.docx]

Supplementary Table 2 (S2) : Detailed neurological examination findings by sex and age group

|  | **Female** | | | | **Male** | | | | |
| --- | --- | --- | --- | --- | --- | --- | --- | --- | --- |
| **Age (years)** | **< 5**  N = 4^1^ | **[5 ; 18)**  N = 296^1^ | **[18 ; 60]**  N = 271^1^ | **> 60**  N = 9^1^ | **< 5**  N = 3^1^ | **[5 ; 18)**  N = 18^1^ | **[18 ; 60]**  N = 60^1^ | **> 60**  N = 8^1^ |  |
| **Head trauma** | 0 (0%) | 107 (36%) | 111 (41%) | 3 (33%) | 1 (33%) | 10 (56%) | 24 (40%) | 2 (25%) |  |
| **Loss of consciousness** | 0 (0%) | 17 (5.7%) | 22 (8.1%) | 1 (11%) | 0 (0%) | 1 (5.6%) | 6 (10%) | 1 (13%) |  |
| **Head Injury Severity Score (0-4)** |  |  |  |  |  |  |  |  |  |
| 0 | 4 (100%) | 189 (64%) | 162 (60%) | 6 (67%) | 2 (67%) | 8 (44%) | 37 (62%) | 6 (75%) |  |
| 1 | 0 (0%) | 90 (30%) | 88 (32%) | 0 (0%) | 1 (33%) | 9 (50%) | 17 (28%) | 1 (13%) |  |
| 2 | 0 (0%) | 14 (4.7%) | 19 (7.0%) | 1 (11%) | 0 (0%) | 1 (5.6%) | 6 (10%) | 1 (13%) |  |
| 3 | 0 (0%) | 3 (1.0%) | 1 (0.4%) | 1 (11%) | 0 (0%) | 0 (0%) | 0 (0%) | 0 (0%) |  |
| 4 | 0 (0%) | 0 (0%) | 1 (0.4%) | 1 (11%) | 0 (0%) | 0 (0%) | 0 (0%) | 0 (0%) |  |
| **Specific head findings** |  |  |  |  |  |  |  |  |  |
| Skull fracture | 0 (0%) | 1 (0.3%) | 0 (0%) | 2 (22%) | 0 (0%) | 0 (0%) | 0 (0%) | 0 (0%) |  |
| Contusion | 0 (0%) | 18 (6.1%) | 13 (4.8%) | 2 (22%) | 0 (0%) | 1 (5.6%) | 6 (10%) | 0 (0%) |  |
| Scalp laceration | 0 (0%) | 4 (1.4%) | 1 (0.4%) | 1 (11%) | 0 (0%) | 0 (0%) | 0 (0%) | 0 (0%) |  |
| Depressed skull fracture | 0 (0%) | 0 (0%) | 0 (0%) | 0 (0%) | 0 (0%) | 0 (0%) | 0 (0%) | 0 (0%) |  |
| Intracranial hemorrhage | 0 (0%) | 2 (0.7%) | 2 (0.7%) | 1 (11%) | 0 (0%) | 0 (0%) | 0 (0%) | 0 (0%) |  |
| **Anatomical location of head injury** |  |  |  |  |  |  |  |  |  |
| Frontal | – | 1 (13%) | 1 (20%) | 0 (0%) | – | 0 (0%) | 1 (100%) | – |  |
| Occipital | – | 5 (63%) | 1 (20%) | 1 (50%) | – | 0 (0%) | 0 (0%) | – |  |
| Temporal | – | 2 (25%) | 3 (60%) | 0 (0%) | – | 1 (100%) | 0 (0%) | – |  |
| Petrous bone | – | 0 (0%) | 0 (0%) | 1 (50%) | – | 0 (0%) | 0 (0%) | – |  |
| Missing | 4 | 288 | 266 | 7 | 3 | 17 | 59 | 8 |  |
| **Facial trauma** | 0 (0%) | 15 (5.1%) | 18 (6.6%) | 3 (33%) | 0 (0%) | 4 (22%) | 5 (8.3%) | 0 (0%) |  |
| **Facial Injury Severity Score (0-2)** |  |  |  |  |  |  |  |  |  |
| 0 | 4 (100%) | 279 (94%) | 251 (93%) | 6 (67%) | 3 (100%) | 14 (78%) | 55 (92%) | 8 (100%) |  |
| 1 | 0 (0%) | 12 (4.1%) | 20 (7.4%) | 2 (22%) | 0 (0%) | 3 (17%) | 3 (5.0%) | 0 (0%) |  |
| 2 | 0 (0%) | 5 (1.7%) | 0 (0%) | 1 (11%) | 0 (0%) | 1 (5.6%) | 2 (3.3%) | 0 (0%) |  |
| **Fracture** | 0 (0%) | 5 (1.7%) | 2 (0.7%) | 1 (11%) | 0 (0%) | 1 (5.6%) | 2 (3.3%) | 0 (0%) |  |
| **Contusion** | 0 (0%) | 13 (4.4%) | 13 (4.8%) | 3 (33%) | 0 (0%) | 4 (22%) | 5 (8.3%) | 0 (0%) |  |
| **Laceration** | 0 (0%) | 4 (1.4%) | 11 (4.1%) | 2 (22%) | 0 (0%) | 2 (11%) | 3 (5.0%) | 0 (0%) |  |
| ^1^n (%) | | | | | | | | | |
