# Supplementary material for: Characteristics of horse riding-related falls in patients presenting to emergency departments in manche department, france: a multicenter retrospective analysis
Source: Scand J Trauma Resusc Emerg Med. 2026 Mar 10;34:76. doi: 10.1186/s13049-026-01593-6 (PMC13088590; doi:10.1186/s13049-026-01593-6)
Supplement: Supplementary file 3 — Supplementary Material 3. [file 13049_2026_1593_MOESM3_ESM.docx]

Supplementary Table 3 (S3): Detailed spinal examination findings by sex and age group

|  | **Female** | | | | **Male** | | | |
| --- | --- | --- | --- | --- | --- | --- | --- | --- |
| **Age (years)** | **< 5**  N = 4^1^ | **[5 ; 18)**  N = 296^1^ | **[18 ; 60]**  N = 271^1^ | **> 60**  N = 9^1^ | **< 5**  N = 3^1^ | **[5 ; 18)**  N = 18^1^ | **[18 ; 60]**  N = 60^1^ | **> 60**  N = 8^1^ |
| **Cervical spine severity (0-4)** |  |  |  |  |  |  |  |  |
| 0 | 4 (100%) | 248 (84%) | 218 (80%) | 7 (78%) | 2 (67%) | 16 (89%) | 55 (92%) | 8 (100%) |
| 1 | 0 (0%) | 44 (15%) | 49 (18%) | 2 (22%) | 1 (33%) | 2 (11%) | 5 (8.3%) | 0 (0%) |
| 2 | 0 (0%) | 2 (0.7%) | 3 (1.1%) | 0 (0%) | 0 (0%) | 0 (0%) | 0 (0%) | 0 (0%) |
| 3 | 0 (0%) | 1 (0.3%) | 1 (0.4%) | 0 (0%) | 0 (0%) | 0 (0%) | 0 (0%) | 0 (0%) |
| 4 | 0 (0%) | 1 (0.3%) | 0 (0%) | 0 (0%) | 0 (0%) | 0 (0%) | 0 (0%) | 0 (0%) |
| **Cervical findings** |  |  |  |  |  |  |  |  |
| Fracture | 0 (0%) | 1 (0.3%) | 2 (0.7%) | 0 (0%) | 0 (0%) | 0 (0%) | 0 (0%) | 0 (0%) |
| Contusion | 0 (0%) | 33 (11%) | 37 (14%) | 2 (22%) | 1 (33%) | 2 (11%) | 5 (8.3%) | 0 (0%) |
| Skin laceration | 0 (0%) | 0 (0%) | 0 (0%) | 0 (0%) | 0 (0%) | 0 (0%) | 0 (0%) | 0 (0%) |
| Sprain | 0 (0%) | 16 (5.4%) | 17 (6.3%) | 0 (0%) | 0 (0%) | 0 (0%) | 0 (0%) | 0 (0%) |
| Dislocation | 0 (0%) | 1 (0.3%) | 0 (0%) | 0 (0%) | 0 (0%) | 0 (0%) | 0 (0%) | 0 (0%) |
| **Thoracic (dorsal) spine severity (0-3)** |  |  |  |  |  |  |  |  |
| 0 | 4 (100%) | 273 (92%) | 253 (93%) | 9 (100%) | 3 (100%) | 17 (94%) | 56 (93%) | 7 (88%) |
| 1 | 0 (0%) | 17 (5.7%) | 12 (4.4%) | 0 (0%) | 0 (0%) | 1 (5.6%) | 1 (1.7%) | 1 (13%) |
| 2 | 0 (0%) | 3 (1.0%) | 5 (1.8%) | 0 (0%) | 0 (0%) | 0 (0%) | 3 (5.0%) | 0 (0%) |
| 3 | 0 (0%) | 3 (1.0%) | 1 (0.4%) | 0 (0%) | 0 (0%) | 0 (0%) | 0 (0%) | 0 (0%) |
| **Thoracic findings** |  |  |  |  |  |  |  |  |
| Fracture | 0 (0%) | 6 (2.0%) | 6 (2.2%) | 0 (0%) | 0 (0%) | 0 (0%) | 3 (5.0%) | 0 (0%) |
| Contusion | 0 (0%) | 18 (6.1%) | 12 (4.4%) | 0 (0%) | 0 (0%) | 1 (5.6%) | 1 (1.7%) | 1 (13%) |
| Skin laceration | 0 (0%) | 0 (0%) | 0 (0%) | 0 (0%) | 0 (0%) | 0 (0%) | 0 (0%) | 0 (0%) |
| Sprain | 0 (0%) | 0 (0%) | 0 (0%) | 0 (0%) | 0 (0%) | 0 (0%) | 0 (0%) | 0 (0%) |
| Dislocation | 0 (0%) | 0 (0%) | 0 (0%) | 0 (0%) | 0 (0%) | 0 (0%) | 0 (0%) | 0 (0%) |
| **Lumbar spine severity (0-3)** |  |  |  |  |  |  |  |  |
| 0 | 4 (100%) | 242 (82%) | 207 (76%) | 8 (89%) | 3 (100%) | 16 (89%) | 50 (83%) | 6 (75%) |
| 1 | 0 (0%) | 48 (16%) | 45 (17%) | 1 (11%) | 0 (0%) | 2 (11%) | 6 (10%) | 1 (13%) |
| 2 | 0 (0%) | 6 (2.0%) | 13 (4.8%) | 0 (0%) | 0 (0%) | 0 (0%) | 2 (3.3%) | 1 (13%) |
| 3 | 0 (0%) | 0 (0%) | 6 (2.2%) | 0 (0%) | 0 (0%) | 0 (0%) | 2 (3.3%) | 0 (0%) |
| **Lumbar findings** |  |  |  |  |  |  |  |  |
| Fracture | 0 (0%) | 5 (1.7%) | 19 (7.0%) | 0 (0%) | 0 (0%) | 0 (0%) | 4 (6.7%) | 1 (13%) |
| Contusion | 0 (0%) | 49 (17%) | 47 (17%) | 1 (11%) | 0 (0%) | 2 (11%) | 7 (12%) | 1 (13%) |
| Skin laceration | 0 (0%) | 0 (0%) | 0 (0%) | 0 (0%) | 0 (0%) | 0 (0%) | 0 (0%) | 0 (0%) |
| Sprain | 0 (0%) | 1 (0.3%) | 0 (0%) | 0 (0%) | 0 (0%) | 0 (0%) | 0 (0%) | 0 (0%) |
| Dislocation | 0 (0%) | 0 (0%) | 0 (0%) | 0 (0%) | 0 (0%) | 0 (0%) | 0 (0%) | 0 (0%) |
| **Fracture pattern (any spine)** |  |  |  |  |  |  |  |  |
| Simple | – | 6 (100%) | 10 (91%) | – | – | – | 4 (67%) | 1 (100%) |
| Displaced | – | 0 (0%) | 0 (0%) | – | – | – | 2 (33%) | 0 (0%) |
| Comminuted | – | 0 (0%) | 1 (9.1%) | – | – | – | 0 (0%) | 0 (0%) |
| Missing | 4 | 290 | 260 | 9 | 3 | 18 | 54 | 7 |
| ^1^n (%) | | | | | | | | |
